# Barriers to Occupational and Physical Therapy Utilization for Children With Cancer

**DOI:** 10.1002/cam4.71598

**Published:** 2026-03-19

**Authors:** Maria C. Swartz, Shiming Zhang, Donna Kelly, Clark R. Andersen, Keri Schadler, Alakh P. Rajan, Eduardo Gonzalez Villarreal, Stephanie J. Wells, Amy Heaton, Karen Moody

**Affiliations:** ^1^ Division of Pediatrics The University of Texas MD Anderson Cancer Center Houston Texas USA; ^2^ Department of Nutrition Sciences and Health Behavior The University of Texas Medical Branch Galveston Texas USA; ^3^ Department of Biostatistics and Data Science UT Health School of Public Health Houston Texas USA; ^4^ Department of Rehabilitation Services The University of Texas MD Anderson Cancer Center Houston Texas USA; ^5^ Department of Biostatistics The University of Texas MD Anderson Cancer Center Houston Texas USA

**Keywords:** cancer, child, occupational therapy, physical therapy, rehabilitation, survivor

## Abstract

**Introduction:**

Occupational and physical therapy (OT/PT) referrals and utilization barriers for childhood cancer patients have not been adequately examined. The current study investigated factors influencing referrals to and utilization of OT/PT services among children with cancer at an NIH‐designated cancer center.

**Methods:**

This retrospective cohort study included pediatric cancer patients (up to 18.99 years) presenting to the center over a 33.5‐month period. Variables that could influence referrals to OT/PT were extracted from the electronic medical record (EMR) including OT and PT referrals (inpatient and outpatient), OT and PT consult completion, age at diagnosis, cancer type, sex, race/ethnicity, insurance, cancer treatment, comorbidities, and social vulnerability index. Mixed‐effect logistic regression models were applied.

**Results:**

The cohort included 1080 patients (mean age 10.9 years; 55.1% male; 40.7% non‐Hispanic White; 52.0% privately insured; 40.8% diagnosed with a non‐neural solid tumor). Overall, 55.1% of inpatients and 22.5% of outpatients were referred for OT/PT, and 32.6% (93.6% of referrals) of inpatients and 13.7% (60.9% of referrals) of outpatients completed their OT/PT consults. Patients aged 18 to < 19 years at diagnosis had lower odds of inpatient OT/PT referral, and patients who underwent surgeries involving hardware placement or had conditions affecting mobility and function, pain, or cardiopulmonary diseases had higher odds of inpatient OT/PT referral. Compared with children with leukemia or lymphoma, children with central nervous system tumors had higher odds of outpatient OT/PT referral, and a non‐neural solid tumor diagnosis was associated with greater odds of outpatient rehabilitation utilization.

**Conclusion:**

Barriers to rehabilitation included age ≥ 18 years, outpatient setting, and leukemia diagnosis. More research is needed to identify strategies, such as using patient navigation and automated referrals, to improve utilization of OT/PT services for the pediatric cancer population.

## Graphical abstract

Significant barriers to occupational and physical therapy (OT/PT) referral and utilization were found among children with cancer, specifically age ≥ 18 years, outpatient setting, and leukemia diagnosis. Addressing these barriers through system‐level interventions is essential to improve utilization of OT/PT services for this population.

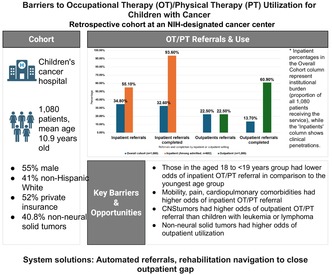

## 1 Introduction

Therapeutic trials and advances in cancer treatment have led to significant improvements in survival rates for children with cancer over the past few decades [1]. However, childhood cancer survivors often experience treatment‐related morbidities that negatively impact their quality of life and physical function and contribute to early frailty [2]. When compared with their healthy siblings, childhood cancer survivors exhibit diminished performance in all areas of physical function [3]. These adverse outcomes can hinder their transition into adult roles, independence in personal care, development of vocational skills, and growth in social responsibility [4].

Childhood cancer survivors tend to lead more sedentary lifestyles compared with their healthy peers, as a consequence of cancer treatment‐related comorbidities, and this is a risk factor for further deconditioning and chronic health issues [3, 5]. Among adult survivors of childhood cancer, 62% will experience a chronic health condition, with 28% facing severe, life‐threatening conditions. These chronic health problems disproportionately impact individuals from underserved racial and ethnic backgrounds, lower income brackets, lower educational levels, and disadvantaged neighborhoods, as well as those without health insurance [6]. There is a critical need for interventions aimed at preventing, reducing, or delaying the onset of chronic health conditions, especially for populations at higher risk [2].

Tailored physical activity and rehabilitation, including occupational therapy (OT) and physical therapy (PT) during cancer treatment, can reduce functional impairments, alleviate chronic health conditions, and promote healthy lifestyles [7]. In this paper, rehabilitation refers specifically to PT and OT, delivered during or after cancer treatment to address treatment‐related functional impairments and promote functional recovery. In children, rehabilitation during cancer therapy has been shown to encourage physical activity [8], and functional independence [9]; maintain strength, endurance, and conditioning; and improve quality of life and mitigate cancer‐related fatigue [10]. Similarly, inpatient rehabilitation after cancer treatment improves quality of life [11], physical activity, strength, and balance [12] and enhances physical, psychosocial, and school functioning [13]. A Cochrane review of children with cancer found that rehabilitation within 5 years of diagnosis significantly improved cardiorespiratory fitness, bone mineral density, and muscle strength [14] Moreover, the National Institutes of Health (NIH) recently endorsed comprehensive rehabilitation services for cancer patients and survivors throughout treatment to improve treatment tolerance and functional outcomes [15].

However, referrals for and utilization of OT and PT services remain insufficient for pediatric cancer patients and survivors [16]. Among over 200 Children's Oncology Group institutions in the United States, only 24% reported having an established pediatric oncology rehabilitation program [16]. A large retrospective cohort study of more than 5000 children with leukemia showed that fewer than 30% received PT within the first year after diagnosis [17], and in a prospective study of over 9000 childhood cancer survivors, less than 9.2% used PT [18].

Data on system‐level barriers to referrals for OT/PT services for pediatric cancer patients are limited. Additionally, it remains unclear how these barriers disproportionately affect vulnerable pediatric populations (e.g., Hispanic and Black populations) and interact with other social determinants of health. The current study aimed to identify system‐level barriers to OT and PT access for children during and after cancer treatment at an NIH‐designated cancer center, specifically examining variables such as clinical practice setting, insurance plan, and interaction of these variables with social determinants of health. We hypothesized that these modifiable system‐level factors would contribute to inconsistencies in care and, by clearly outlining these factors, we could identify opportunities to improve access to rehabilitation for all children with cancer. Recognizing that access to rehabilitation services for children with cancer is influenced by factors operating at multiple levels, we built on the ecological systems framework [19, 20]. The ecological systems framework states that access to and use of healthcare services are based on a complex interplay of factors including individual, community, organizational, and policy‐level influences. We conceptualized barriers and facilitators drawn from previous studies [21, 22], system‐level barriers to OT and PT access for children during and after cancer treatment at an NIH‐designated cancer center. Specifically, we examined the association between system‐level factors (e.g., clinical care setting and insurance type) and OT and PT referrals, while accounting for individual‐level and clinical‐level factors. We hypothesized that system‐level factors (e.g., clinical care setting and insurance type) would contribute to inconsistencies in care. By identifying specific system‐level barriers, we could then identify opportunities to improve access for all children with cancer.

## 2 Materials & Methods

In this retrospective cohort study, OT and PT data were extracted from the electronic medical record (EMR) using structured data fields. Rehabilitation referrals (access) were identified through provider‐initiated referral orders coded as “occupational therapy” or “physical therapy” within the EMR order entry system. Completion (utilization) of OT and PT was determined based on documentation of at least one corresponding OT or PT encounter, visit note, or billing code within the same episode of care (admission) or outpatient. All extracted data were verified by cross‐checking against therapy encounter logs provided by the Rehabilitation Department to ensure accuracy. Because OT/and PT referrals are standardized and documented in discrete EMR fields at our institution, variability in documentation was minimal compared with other supportive care services, which may be captured in unstructured notes. For the purposes of this study, rehabilitation was operationally defined as receipt of occupational therapy (OT) and/or physical therapy (PT) services documented within the EMR. Other domains of rehabilitation care (e.g., speech‐language pathology, neuropsychology, or recreational therapy) were not included in this analysis.

### 2.1 Study Cohort Collection

Our study included all pediatric patients and survivors with at least 1 completed visit to medical professionals in the Department of Pediatrics between March 6, 2016 (when the institution transitioned to the EPIC system) and December 31, 2019 (prior to the COVID‐19 pandemic), and who were diagnosed with cancer before the age of 19 years.

To ensure accuracy, we validated all variables (cancer diagnosis, diagnosis date, medical record number) by cross‐referencing the institution's tumor registry with the EMR system, which includes both inpatient and outpatient data. We excluded children who lacked a confirmed cancer diagnosis or had only one visit at our institution. The step‐by‐step selection process is outlined in Table 1.

**TABLE 1:** Steps in Pediatric Cancer Cohort Selection (Data Sources: Institutional Tumor Registry and Electronic Medical Record System).

Step	Selection criteria	No. of eligible patients
Step 1	All patients treated in the Department of Pediatrics from March 6, 2016, to December 31, 2019. This time frame aligned with the EMR launch at our institution and avoided the COVID19 pandemic which interrupted normal operations.	1335
Step 2	Excluded patients with no record of age at cancer diagnosis or those with < 2 visits, to avoid one‐time consultations (*n* = 92)	1243
Step 3	Excluded patients with a childhood cancer diagnosis who were older than 19 years at the time of diagnosis (*n* = 139)	1104
Step 4	Validation of extracted data: excluded patients without confirmed cancer (e.g., benign diagnoses) at all time points (*n* = 24)	1080

### 2.2 Variables of Interest

Variables were selected based on empirical support from literature or clinical relevance to referrals to and utilization of OT/PT services. These variables reflect the complexity of. accessing and utilizing cancer care, including OT/PT care. We chose this approach to examine how barriers and facilitators at the system/community‐level affect referrals and utilization while accounting for individual‐level and clinical‐level factors. Table S1 provides the rationale for the selected variables. Individual/patient‐level factors included demographic data such as age at diagnosis, sex, race/ethnicity, and language spoken [23, 24, 25]. The disease/clinical‐level factor included cancer diagnosis, age at diagnosis, cancer treatments (chemotherapy and/or radiation therapy), surgical procedures, and comorbidities [26, 27]. Cancer diagnoses, surgical procedures, and comorbidities were independently reviewed and re‐categorized by two clinical team members (KM, DK) for data analysis. Specifically, categories were developed by KM to combine the data for each of the headings of cancer diagnoses, surgical procedures, and comorbidities based on clinical relevance. Then, KM assigned a category to each discrete data element and DK reviewed the categorization for confirmation until all data was categorized. Disagreements were handled by consensus. Cancer diagnoses were reclassified from 383 categories into four groups: non‐neural solid tumors, central nervous system (CNS) tumors, leukemia/lymphoma, and adult‐type cancer (e.g., breast, thyroid, melanoma). Comorbidities were also reclassified using the same process as above from 4188 categories into five groups to facilitate data analyses. These groups included the following: (1) cardiopulmonary diseases; (2) conditions affecting mobility and function, including neurological diseases, orthopedic diseases, and psychiatric disorders; (3) acute and chronic pain conditions; (4) diseases and conditions affecting other organ systems; and (5) non‐disease diagnoses such as “family history of genetic disease” or “influenza vaccine recommended.” Surgical procedures were reclassified from 1075 categories into five groups: (1) amputations and tumor resections, (2) diagnostic procedures (e.g., biopsy, lumbar puncture), (3) cancer‐related surgeries and procedures (e.g., exploratory laparotomy, skin graft procedure), (4) placement or removal of hardware/medical devices (e.g., gastric tube placement), (5) other non–cancer‐related surgeries/procedures (e.g., tooth extraction, hernia repair). Differences in classification were handled by consensus. Lastly, the system/community‐level factors included care setting (inpatient or outpatient), insurance type (private, Medicaid, or other), and Social Vulnerability Index [23, 25, 26, 27, 28, 29]. Social vulnerability index is a census tract‐level measure developed by the Centers for Disease Control and Prevention that quantifies neighborhood disadvantage. The percentile ranking identifies communities experiencing compounded social disadvantages. This variable was included to account for potential disparities in referrals and utilization due to social factors that were not available within the EMR. Higher SVI means higher vulnerability [28].

### 2.3 Outcomes of Interest

The primary outcome of interest was the prevalence of cancer OT/PT service referrals in both inpatient and outpatient settings. Secondary outcomes included the completion of cancer rehabilitation service (i.e., actual utilization of OT and/or PT services) in these same settings and predictors of referrals and utilization of cancer rehabilitation service in both settings.

### 2.4 Statistical Analysis

The frequency of pediatric inpatient and outpatient OT/PT referrals was summarized using descriptive statistics, indicating whether each patient had been referred at least once. Similarly, the prevalence of completed inpatient and outpatient OT/PT referrals was calculated, reflecting the proportion of referred patients who successfully completed the service at least once.

All data were validated prior to analysis. Univariate mixed‐effects logistic regression models were used to examine the relationship between the four outcome variables (inpatient referral status, inpatient referral completion status, outpatient referral status, and outpatient referral completion status) and each demographic and clinical characteristic. Cancer treatments, including chemotherapy, radiation therapy, and cancer surgical resection, were represented as binary variables indicating whether patients had ever received these treatments.

The univariate mixed‐effects logistic regression models were used to identify factors associated with OT/PT referrals and their completion status in both inpatient and outpatient settings, and these factors were then incorporated into the multivariable models. Covariates for the multivariable models were selected based on a combination of low Akaike Information Criterion, clinical relevance, and noncollinearity. A common set of variables was used across all multivariable models. These covariates included sex, race/ethnicity, cancer diagnosis category (leukemia/lymphoma, non‐neural solid tumor, CNS tumor, or adult‐type cancer), presence of radiation therapy plan, primary insurance coverage (Medicaid, private, or nonprivate/other/government‐issued/unknown), presence of chemotherapy plan, presence of hormonal therapy plan, Social Vulnerability Index percentile, and presence of comorbidities, including conditions affecting mobility and function, cardiopulmonary diseases, and diseases and conditions affecting other organ systems. Noncollinearity was confirmed with variance inflation factors of less than 2 for all four multivariable models. Given the moderate sample size [17] we prioritized stable main effects models for the current study.

Differences among levels of nonbinary discrete covariates were assessed using Dunnett‐adjusted contrasts, with comparisons made to the reference category. A 95% confidence level was applied for all statistical tests. Statistical analyses were conducted using RStudio software version 4.4.0, RStudio Team 2024.

## 3 Results

### 3.1 Cancer Patient Cohort

The final cohort consisted of 1080 pediatric cancer patients. The average age at cancer diagnosis was 10.9 years (range 0–18.9 years; SD 5.6). Over half of the cohort (55.1%) was male, 40.7% were non‐Hispanic White, 74.4% spoke English, and 52.0% had private primary insurance coverage. The mean Social Vulnerability Index percentile (on a scale from 0 to 100) was 57.96 (SD 29.60).

In terms of clinical characteristics, 441 patients (40.8%) were diagnosed with a non‐neural solid tumor such as osteosarcoma or rhabdomyosarcoma. Almost all pediatric cancer patients received chemotherapy (98.5%), and 62.8% had at least one comorbidity. These comorbidities included conditions affecting mobility and function (32.1%), acute and chronic pain conditions (37.1%), cardiopulmonary diseases (29.6%), and diseases and conditions affecting other organ systems (62.5%). Additionally, 71.0% of patients underwent some type of procedure, with 50.4% undergoing placement or removal of hardware/medical devices. Table 2 provides a summary of the demographic and clinical characteristics of our study cohort.

**TABLE 2:** Demographic and Clinical Characteristics of the Pediatric Cancer Patient Cohort (*n* = 1080).

Variable	No. (%)
Age at cancer diagnosis	
Mean (SD)	10.9 years (5.6 years)
Range	0.00–18.9 years
0.1–5 years	272 (25.2)
6–9	188 (17.4)
10–13 years	182 (16.9)
14–17 years	338 (31.3)
≥ 18 years	100 (9.3)
Sex	
Female	485 (44.9)
Male	595 (55.1)
Language spoken	
English	804 (74.4)
Spanish	170 (15.7)
Other	106 (9.8)
Race/Ethnicity	
Hispanic	314 (29.1)
Non‐Hispanic Asian	90 (8.3)
Non‐Hispanic black	91 (8.4)
Non‐Hispanic white	440 (40.7)
Other/Unknown/Declined	145 (13.4)
Primary insurance coverage	
Medicaid	303 (28.1)
Medicare	5 (0.5)
Nonprivate/Other/Government‐Issued/Unknown	215 (19.9)
Private	562 (52.0)
Chemotherapy plan	
Yes	1064 (98.5)
No	16 (1.5)
Hormonal therapy plan	
Yes	47 (4.4)
No	592 (54.8)
Missing	441 (40.8)
Radiation therapy plan	
Yes	262 (24.3)
No	818 (75.7)
Cancer type	
Leukemia/Lymphoma	270 (25.0)
Non‐neural solid tumor	441 (40.8)
Central nervous system tumor	297 (27.5)
Adult‐type cancer	72 (6.7)
More than one cancer diagnosis	19 (1.8)
Procedures performed	
No Procedure	313 (29.0)
Any Procedure	767 (71.0)
Specific procedures^a^	
Diagnostic Procedures	272 (25.2)
Placement or Removal of Hardware/Medical Devices	544 (50.4)
Cancer‐Related Surgeries and Procedures	304 (28.1)
Amputations and Tumor Resections	92 (8.5)
Other Non–Cancer‐Related Surgeries/Procedures	16 (1.5)
Social Vulnerability Index Overall Percentile	
Mean (SD)	57.96 (29.60)
Range	0.05–99.94
Admitted to Inpatient Unit	
Yes	682 (63.1)
No	398 (36.9)
Comorbidities^b^	
Conditions Affecting Mobility and Function	347 (32.1)
Acute and Chronic Pain Conditions	401 (37.1)
Cardiopulmonary Diseases	320 (29.6)
Diseases and Conditions Affecting Other Organ Systems	675 (62.5)
Non‐Disease Diagnoses	516 (47.8)

^a^ (1) Diagnostic procedures includes biopsy, lumbar puncture, etc., (2) placement or removal of hardware/medical devices (e.g., gastric tube placement), (3) cancer‐related surgeries and procedures (e.g., exploratory laparotomy, skin graft procedure), (4) amputations and tumor resections, (5) other non–cancer‐related surgeries/procedures (e.g., tooth extraction, hernia repair).

^b^ Some patients had > 1 comorbidity.

### 3.2 Referrals and Utilization of Inpatient and Outpatient OT/PT

In our cohort of 1080 patients, 682 were admitted to the inpatient wards at least once. Of these, 376 patients (55.1% of the admitted patients, or 34.8% of the entire cohort) received at least one inpatient OT/PT referral. Additionally, 352 patients (51.6% of the admitted patients) completed at least one inpatient OT/PT consultation, representing 32.6% of the entire cohort. Among referred patients, median age at referral was 12.6 years, ranging from 3 months to 24 years, with a median of 3 months between diagnosis and referral, ranging from 0 to 20 years. Of the 376 patients who received an inpatient referral, 352 patients (93.6%) successfully completed the referral. Of the overall cohort, 243 patients (22.5%) were referred to outpatient OT/PT, and 148 of these patients (60.9%) completed their outpatient OT/PT consultation, representing 13.7% of the entire cohort (Tables 3, and S2). Successful completion of an OT or PT referral was operationally defined as attending at least one therapy session following the referral, as documented in the EMR through encounter notes or billing records. Tables S3–S6 show results of the univariate analyses for inpatient and outpatient OT/PT referrals and utilization.

**TABLE 3:** Inpatient and Outpatient Referrals to Occupational or Physical Therapy and Completion Status.

Primary outcome	No. (%)		
	Overall cohort, *n* = 1080	Inpatients (among admitted), *n* = 682	Outpatients, *n* = 1080
Inpatient referrals	376 (34.8)^a^	376 (55.1)	—
Inpatient referrals completed	352 (32.6)^a^	352/376 (93.6)	—
Outpatient referrals	243 (22.5)	—	243 (22.5)
Outpatient referrals completed	148 (13.7)	—	148/243 (60.9)

^a^ Inpatient percentages in the Overall Cohort column represent institutional burden (proportion of all 1080 patients receiving the service), while the ‘Inpatients’ column shows clinical penetrations. The difference reflects that 398 patients (36.9%) were never admitted and thus may potentially never have an opportunity to be referred to OT/PT services, regardless of clinical need.

### 3.3 Inpatient Referrals and Utilization of OT/PT

The multivariable model showed that age at diagnosis, 18 to < 19 years, was associated with significantly lower odds of receiving inpatient OT/PT referrals compared with the youngest age group (0–9 years; OR 0.1156, 95% CI 0.0457–0.2927, *p* < 0.0001). In addition, those who underwent “other non–cancer‐related surgeries/procedures” had significantly lower odds of receiving an inpatient OT/PT referral (OR 0.4292, 95% CI 0.1891–0.9742, *p* = 0.043). Other significant positive predictors of inpatient referral to OT/PT included surgery involving placement or removal of hardware/medical devices and the following comorbidities: conditions affecting mobility and function, acute and chronic pain conditions, and cardiopulmonary diseases (Table 4). The multivariable model did not yield any significant risk factors associated with completion of inpatient OT/PT services (Table 4).

**TABLE 4:** Multivariable Analysis of Factors Associated with Inpatient Referrals to Occupational or Physical Therapy (Referrals) and Completion of Services (Utilization).

Covariate	Contrast	Odds Ratio (95% CI)	*p* *
Referrals			
Age at diagnosis, discrete	6–9/0.1–5 years	1.2196 (0.5715, 2.6028)	0.93
	10–13/0.1–5 years	1.3125 (0.5998, 2.8719)	0.86
	14–17/0.1‐ 5 years	1.0399 (0.5274, 2.0503)	1
	18 to < 19/0.1–5 years	0.1259 (0.0472, 0.3361)	**0.0001**
Sex	Male/Female	0.8854 (0.5307, 1.4772)	0.64
Race/Ethnicity	Hispanic/Non‐Hispanic White	0.8223 (0.4457, 1.5172)	0.88
	Non‐Hispanic Black/Non‐Hispanic White	0.878 (0.3887, 1.9833)	0.98
	Non‐Hispanic Asian/Non‐Hispanic White	1.1625 (0.3552, 3.8046)	0.99
	(Other/Unknown/Declined)/Non‐Hispanic White	0.8675 (0.3733, 2.0159)	0.97
Primary Insurance Coverage	Private/Medicaid	1.5228 (0.8298, 2.7945)	0.30
	(Nonprivate/Other/Government‐Issued/Unknown)/Medicaid	0.6122 (0.2179, 1.7196)	0.55
Chemotherapy Plan	Yes/No	8.6229 (0.7092,104.842)	0.09
Hormonal Therapy Plan	Yes/No	1.4377 (0.5276, 3.9174)	0.48
Radiation Therapy Plan	Yes/No	1.2772 (0.6708, 2.4321)	0.46
Cancer Type	NNST/(Leukemia/Lymphoma)	1.4144 (0.8096, 2.4711)	0.47
	CNS Tumor/(Leukemia/Lymphoma)	1.4536 (0.6465, 3.2681)	0.67
	Adult‐Type Cancer/(Leukemia/Lymphoma)	0.6713 (0.2216, 2.0334)	0.79
Procedure – Diagnostic Procedures	Yes/No	1.3734 (0.6808, 2.7707)	0.38
Procedure – Placement or Removal of Hardware/Medical Devices	Yes/No	2.2524 (1.0499, 4.8323)	**0.037**
Procedure—other non–cancer‐related surgeries/procedures	Yes/No	0.4334 (0.1906, 0.9858)	**0.046**
SVI overall percentile	Unit change	1.007 (0.9979, 1.0161)	0.13
Comorbidities—conditions affecting mobility and function	Yes/No	1.8563 (1.1129, 3.0962)	**0.018**
Comorbidities—acute and chronic pain conditions	Yes/No	1.7452 (1.0128, 3.0071)	**0.045**
Comorbidities – Cardiopulmonary Diseases	Yes/No	2.8867 (1.7327, 4.8095)	**< 0.0001**
Comorbidities – Diseases and Conditions Affecting Other Organ Systems	Yes/No	1.403 (0.8048, 2.4456)	0.23
Utilization			
Age, discrete	6–9/0.1–5 years	0.9545 (0.2128, 4.2818)	1
	10–13/0.1–5 years	0.6509 (0.1744, 2.4294)	0.88
	14–17/0.1–5 years	1.05 (0.2648, 4.1641)	1
	18 to < 19/0.1–5 years	0.6888 (0.047, 10.0984)	0.98
Sex	Male/Female	1.5934 (0.6119, 4.1491)	0.34
Race/Ethnicity	Hispanic/Non‐Hispanic White	0.5111 (0.1616, 1.6163)	0.59
	Non‐Hispanic Black/Non‐Hispanic White	0.8209 (0.1595, 4.225)	0.99
	Non‐Hispanic Asian/Non‐Hispanic White	1.1283 (0.0989, 12.8766)	1
	(Other/Unknown/Declined)/Non‐Hispanic White	0.4224 (0.1022, 1.7451)	0.56
Primary Insurance Coverage	Private/Medicaid	1.6792 (0.5668, 4.9753)	0.55
	(Nonprivate/Other/Government‐Issued/Unknown)/Medicaid	3.6423 (0.2404, 55.1947)	0.55
Chemotherapy Plan	Yes/No	3.8505 (0.0303, 489.3622)	0.59
Hormonal Therapy Plan	Yes/No	3.1508 (0.2929, 33.891)	0.34
Radiation Therapy Plan	Yes/No	0.9182 (0.3119, 2.7033)	0.88
Cancer Type	NNST/(Leukemia/Lymphoma)	0.6204 (0.2028, 1.8973)	0.72
	CNS Tumor/(Leukemia/Lymphoma)	0.2556 (0.0602, 1.0846)	0.17
	Adult‐Type Cancer/(Leukemia/Lymphoma)	0.2732 (0.0377, 1.9803)	0.43
Procedure—Diagnostic Procedures	Yes/No	0.8322 (0.2341, 2.9584)	0.78
Procedure – placement or removal of hardware/medical devices	Yes/No	0.8745 (0.2426, 3.152)	0.84
Procedure – other non–cancer‐related surgeries/procedures	Yes/No	5.1688 (0.4169, 64.0864)	0.20
SVI overall percentile	Unit Change	1.0173 (0.9986, 1.0363)	0.13
Comorbidities – conditions affecting mobility and function	Yes/No	0.8055 (0.2968, 2.1866)	0.67
Comorbidities – acute and chronic pain conditions	Yes/No	0.8092 (0.2732, 2.3971)	0.7
Comorbidities – cardiopulmonary diseases	Yes/No	2.2422 (0.8633, 5.8233)	0.1
Comorbidities – diseases and conditions affecting other organ systems	Yes/No	1.0808 (0.3226, 3.6204)	0.90

Abbreviations: CNS, central nervous system; NNST, non‐neural solid tumor; SVI, Social vulnerability index.

* Boldface indicates statistical significance.

### 3.4 Outpatient Referrals and Utilization of OT/PT

The multivariable model showed that, compared with patients with leukemia/lymphoma, patients with a CNS tumor had significantly higher odds of receiving an outpatient OT/PT referral (OR 3.3242, 95% CI 1.6468–6.710.3, *p* = 0.002). In addition, patients with a comorbid condition affecting mobility or function (OR 4.1159, 95% CI 2.5174–6.7295, *p* < 0.0001) or disease or condition affecting other organ systems (OR 2.0956, 95% CI 1.1959–3.6722, *p* = 0.01) also had significantly higher relative odds of being referred to OT and PT services (Table 5). With respect to OT/PT utilization, patients who had a non‐neural solid tumor diagnosis had significantly higher odds of completing the outpatient OT/PT services compared with patients diagnosed with leukemia/lymphoma (OR 4.3960, 95% CI 1.7331–11.1504, *p* = 0.006; Table 5).

**TABLE 5:** Multivariable Analysis of Factors Associated with Outpatient Referrals to Occupational or Physical Therapy (Referrals) and Completion of Services (Utilization).

Covariate	Contrast	Odds Ratio (95% CI)	*p* *
Referrals			
Age at diagnosis, discrete	6–9/0.1–5 years	1.352 (0.6564, 2.7849)	0.79
	10–13/0.1–5 years	1.4927 (0.7122, 3.1286)	0.64
	14–17/0.1–5 years	0.9412 (0.489, 1.8116)	0.99
	18 to < 19/0.1–5 years	0.7369 (0.299, 1.8159)	0.87
Sex	Male/Female	0.7906 (0.4875, 1.2819)	0.34
Race/Ethnicity	Hispanic/Non‐Hispanic White	1.3217 (0.7387, 2.3649)	0.72
	Non‐Hispanic Black/Non‐Hispanic White	1.1691 (0.5325, 2.5665)	0.96
	Non‐Hispanic Asian/Non‐Hispanic White	1.7223 (0.6225, 4.7654)	0.65
	(Other/Unknown/Declined)/Non‐Hispanic White	1.45 (0.6567, 3.2017)	0.73
Primary Insurance Coverage	Private/Medicaid	0.8498 (0.48, 1.5048)	0.79
	(Nonprivate/Other/Government‐Issued/Unknown)/Medicaid	0.9669 (0.3652, 2.5599)	0.99
Chemotherapy Plan	Yes/No	7.8628 (0.7687, 80.4251)	0.08
Hormonal Therapy Plan	Yes/No	1.7302 (0.7493, 3.9949)	0.2
Radiation Therapy Plan	Yes/No	0.9162 (0.5181, 1.6201)	0.76
Cancer Type	NNST/(Leukemia/Lymphoma)	1.6671 (0.9634, 2.8848)	0.17
	CNS Tumor/(Leukemia/Lymphoma)	3.3961 (1.6755, 6.8836)	**0.002** *
	Adult‐Type Cancer/(Leukemia/Lymphoma)	0.3887 (0.1012, 1.4926)	0.38
Procedure – Diagnostic Procedures	Yes/No	1.9022 (1.0006, 3.6161)	**0.0498** *
Procedure – Placement or Removal of Hardware/Medical Devices	Yes/No	0.7719 (0.3684, 1.6171)	0.49
Procedure – Other Non–Cancer‐Related Surgeries/Procedures	Yes/No	0.6377 (0.2826, 1.4388)	0.28
SVI Overall Percentile	Unit Change	0.9993 (0.9908, 1.0079)	0.87
Comorbidities – Conditions Affecting Mobility and Function	Yes/No	4.0775 (2.4932, 6.6684)	**< 0.0001** *
Comorbidities – Acute and Chronic Pain Conditions	Yes/No	1.6431 (0.9638, 2.8013)	0.068
Comorbidities – Cardiopulmonary Diseases	Yes/No	1.0061 (0.6102, 1.659)	0.98
Comorbidities – Diseases and Conditions Affecting Other Organ Systems	Yes/No	2.0864 (1.1895, 3.6593)	**0.01** *
Utilization			
Age, discrete	6–9/0.1–5 years	0.4182 (0.135, 1.2953)	0.37
	10–13/0.1–5 years	1.1827 (0.3585, 3.902)	0.98
	14–17/0.1–5 years	1.285 (0.4275, 3.8626)	0.95
	18 to < 19/0.1–5 years	0.6127 (0.143, 2.6249)	0.87
Sex	Male/Female	0.4867 (0.2184, 1.0843)	0.08
Race/Ethnicity	Hispanic/Non‐Hispanic White	1.222 (0.4819, 3.0984)	0.95
	Non‐Hispanic Black/Non‐Hispanic White	1.5792 (0.4527, 5.5095)	0.84
	Non‐Hispanic Asian/Non‐Hispanic White	0.7447 (0.1551, 3.5756)	0.97
	(Other/Unknown/Declined)/Non‐Hispanic White	0.3126 (0.0899, 1.0876)	0.21
Primary Insurance Coverage	Private/Medicaid	1.6036 (0.6561, 3.9193)	0.48
	(Nonprivate/Other/Government‐Issued/Unknown)/Medicaid	1.4691 (0.3029, 7.1249)	0.84
Chemotherapy Plan	Yes/No	0.3457 (0.0067, 17.8515)	0.6
Hormonal Therapy Plan	Yes/No	3.555 (0.9326, 13.5509)	0.066
Radiation Therapy Plan	Yes/No	1.0169 (0.3964, 2.6087)	0.97
Cancer Type	NNST/(Leukemia/Lymphoma)	4.208 (1.6566, 10.6891)	**0.009** *
	CNS Tumor/(Leukemia/Lymphoma)	1.9768 (0.6913, 5.6529)	0.44
	Adult‐Type Cancer/(Leukemia/Lymphoma)	1.0342 (0.1003, 10.6602)	1
Procedure – Diagnostic Procedures	Yes/No	0.947 (0.3576, 2.5076)	0.91
Procedure – Placement or Removal of Hardware/Medical Devices	Yes/No	1.6124 (0.4249, 6.1187)	0.48
Procedure – Other Non–Cancer‐Related Surgeries/Procedures	Yes/No	2.2132 (0.5971, 8.2036)	0.24
SVI Overall Percentile	Unit Change	0.9914 (0.9779, 1.005)	0.23
Comorbidities – Conditions Affecting Mobility and Function	Yes/No	2.4756 (1.0025, 6.1132)	0.052
Comorbidities – Acute and Chronic Pain Conditions	Yes/No	0.5075 (0.1894, 1.3598)	0.18
Comorbidities – Cardiopulmonary Diseases	Yes/No	1.0635 (0.4679, 2.4174)	0.88
Comorbidities – Diseases and Conditions Affecting Other Organ Systems	Yes/No	0.4722 (0.164, 1.3593)	0.17

Abbreviations: CNS, central nervous system; NNST, non‐neural solid tumor; SVI, Social Vulnerability Index.

* Significance *p*‐value < ;0.05.

## 4 Discussion

The integration of cancer rehabilitation into comprehensive cancer care is essential to ensuring optimal functional outcomes for pediatric patients with cancer [30, 31] Expert consensus recommendations exist across pediatric oncology diagnoses for rehabilitation referral, assessment, and management [32]. However, adherence to these guidelines remains a challenge [17, 18, 33], and studies examining institutional barriers to rehabilitation referral and utilization rates among pediatric cancer survivors are limited [17, 18, 34]. The current study, conducted at an NIH‐designated cancer center, aimed to identify system‐level barriers to OT/PT care for children during and after cancer treatment, as well as how these barriers may contribute to inconsistencies in care. Our findings aligned with both our hypothesis and the ecological systems framework. In addition to individual and clinical‐level factors, system/community‐level factors such as the clinical setting (inpatient versus outpatient), showed a differential influence in referrals and utilization to OT and PT. Since inpatient and outpatient OT and PT services in the U.S. rely on different referral and authorization processes, there are opportunities to improve the implementation of outpatient OT/PT by modifying the existing procedures used to deliver these services.

Approximately 55% of children in our cohort were referred for and received OT and/or PT during hospitalization, a finding that showed a higher rate than reported for pediatric leukemia patients across 330 hospitals [17]. However, only 22.5% were referred for outpatient OT/PT services, and even fewer (13.7%) received these services. This low outpatient utilization rate is consistent with other research reporting that 25% of children with cancer are referred to rehabilitation [18], as well as data from the Children's Cancer Survivor Study, which reported that 9.2% of childhood cancer survivors received outpatient rehabilitation services [35]. Of note in the Children's Cancer Survivor Study, 20% of the cohort reported physical performance deficits pointing to an unmet need for OT/PT [36, 37]. Compared with inpatient OT and PT referrals, cancer OT and PT referrals in the outpatient setting depend more on provider practice, geographics (patient location of residence), and availability of rehabilitation centers in the community. Furthermore, the need for rehabilitation therapists with expertise in both cancer care and pediatrics narrows the pool of available community providers.^22,24,^

In the current study, multivariable analyses revealed several significant individual‐level and disease/clinical‐level positive and negative predictors of referrals to and utilization of rehabilitation services.

On the inpatient side, age 18 to < 19 years was a negative predictor of rehabilitation, and this may reflect a lower level of clinical need, a pattern of care by specific practices that care for older patients, or adults declining the referral to rehabilitation services. It may also reflect more adults seeking OT/PT care in the community, although we cannot confirm this given our study methods. Similar findings have been revealed by Tanner et al. [38], who noted that the oldest age group in their cohort (> 18 years) consistently received fewer rehabilitation services across settings, and by Ramphal et al. [39], who found that adolescents and young adults often face lower referral rates to supportive care services, including OT/PT.

Positive predictors of inpatient OT and PT included comorbid conditions for which rehabilitation has a strong evidence base (surgery involving placement or removal of hardware/medical devices, acute and chronic pain conditions, conditions affecting mobility and function, and cardiopulmonary diseases) [22]. In addition, inpatients who underwent surgeries and procedures unrelated to cancer were less likely to be referred to OT and PT, suggesting that rehabilitation services may not have been appropriate for this group. The development of programs such as the CREATE initiative by Tanner et al. [38] highlights the importance of clear guidance on when to refer patients to OT/PT services, especially for those undergoing other surgical procedures. Not surprisingly, the completion rate for inpatient OT/PT services at our institution was high, approximately 94%, likely because travel, insurance preauthorization, and identification of a service provider are not required in this setting.

Compared with the inpatient setting, the outpatient setting showed a notably lower rate of referrals to OT/PT, and this finding is consistent with outpatient OT/PT referral gaps reported in the literature [18, 34]. Fortunately, some high‐risk groups were more likely to be referred to OT and PT, such as those with comorbid conditions affecting mobility or function and those with CNS tumors. Children with CNS tumors are at significant risk for functional impairments [40], and research has shown that rehabilitation leads to substantial functional gains in this high‐risk population [41]. The relative increase in referrals to rehabilitation for patients with CNS tumors compared with leukemia/lymphoma patients has also been noted by other groups [42]. This lower rate of rehabilitation referrals for children with leukemia/lymphoma is particularly concerning given the documented benefits of this intervention for these children [43, 44]. Completion rates of outpatient OT/PT referrals also significantly differed by diagnosis and was highest in patients with non‐neural solid tumors. This group includes patients with sarcoma diagnoses, in whom functional impairments may be more visible (e.g., amputations) and thus more likely to drive the completion of OT/PT services. In addition, at our center, OT/PT services are embedded in the orthopedic surgery clinic, which cares for many patients in this group and likely fostered completion of rehabilitation referrals. Based on the literature, additional institutional strategies to increase OT/PT utilization include embedded into a pediatric oncology clinic, and co‐location of rehabilitation space near the clinic to minimize missed appointments due to delays in chemotherapy or transfusion services [38].

Sex, Social Vulnerability Index, insurance, and race/ethnicity did not emerge as predictors of OT and PT referral or utilization in either inpatient or outpatient settings in our cohort. However, the low overall referral and utilization rates for outpatient OT/PT services remain concerning, particularly given the well‐established benefits of cancer rehabilitation in promoting physical activity, independence, and quality of life in this population [7, 10].

Recently, published recommendations tailored to diagnosis and anticipated functional impairments have been published for children with cancer [32]. Promoting adherence to these recommendations could improve outpatient referral patterns and ensure more equitable referrals to rehabilitation across service lines. Additionally, the development of prospective surveillance monitoring programs such as the CREATE initiative by Tanner et al. [38] has been shown to increase utilization rates of OT/PT in children with cancer across diagnoses. In fact, the CREATE program increased both inpatient and outpatient rehabilitation service utilization from rates similar to those shown in the current study to rates exceeding 80%. A key change that occurred as a result of the CREATE program was the automation of rehabilitation referrals within patients' admission orders in the EMR. Given CREATE's success, implementing automated rehabilitation referrals removes reliance on providers in making referral decisions. This would overcome clinician bias, variability in practice, and limitations in bandwidth, therefore averting barriers related to patients' age or clinical disease group. Similarly, automation of all new outpatients for a screening OT/PT visit would ensure that all children in need of OT/PT would be connected to services. Standardizing referral timing would prevent delays in care and ensure all eligible patients are referred, which optimizes care equity [45, 46].

Another potential solution to bridging the gap between oncology and rehabilitation care that can be applied in the outpatient setting is the establishment of a cancer patient navigator role specializing in rehabilitation coordination [15]. This role would involve care coordination, including conducting functional assessments (screenings) and triaging patients for more thorough assessments by the rehabilitation team. For discharging patients, patient navigators would facilitate communication between the oncology and rehabilitation teams and assist with ensuring outpatient referrals were in place, begin the insurance approval processes and ensure identification of available community providers. For outpatients, navigators would follow patients to make sure they do not get lost to follow up when missing OT/PT appointments due to unexpected admissions, blood transfusions, and acute illness. With the approval of new cancer navigation billing codes (Healthcare Common Procedure Coding System codes G0023 and G0024 and Current Procedural Terminology codes 99,424–99,427), the integration of patient navigators could significantly improve referrals to OT and PT services in the outpatient setting.

The current study had some limitations. The findings from this single‐site study at a tertiary academic cancer center with > 50% privately insured patients may not be generalizable to other cancer centers serving a greater number of publicly insured or uninsured patients. Additionally, we did not link patient data to claims data, which could potentially underestimate OT/PT utilization, particularly for patients accessing these services outside our institution and within the community setting. Furthermore, our analysis captured only referral and initial utilization patterns within our EMR; we could not assess sustained engagement because we were unable to reliably align OT/PT referral dates with outpatient visit frequency and duration of service use in the outpatient setting. Future research utilizing institution‐based and claims data is needed to fully characterize rehabilitation engagement. Also of note, our analysis was limited to structured data relating only to OT/PT referrals within the EMR. Unstructured data, such as clinical narrative data on patient function, health status, and non‐medical drivers of health, could provide additional insights into the low referral and utilization rates for outpatient OT/PT services. Moreover, our study only included the assessment of OT and PT referrals. Therefore, we cannot make any claims regarding referrals to other rehabilitative services such as speech/language pathology, recreational therapy, or vocational counseling. Lastly, our combined analysis of OT/PT services reflects co‐referral practice patterns at our institution.

Despite these limitations, our findings point to opportunities for improving referrals to and utilization of OT and PT for children with cancer, especially in the outpatient setting. Improving adherence to current published recommendations for referrals and implementing an automated referral process are actionable steps that oncology providers could take to immediately improve referrals to OT and PT for their patients.

## 5 Conclusion

Our findings reveal significant gaps in referrals to and utilization of OT and PT among children with cancer at our institution, particularly in the outpatient setting. We identified specific patient subsets who were less likely to be referred for these services, clinical service lines that were more likely to provide OT/PT referrals, and diagnoses associated with higher utilization of OT/PT services following referral. Given that OT/PT is a critical component of the quality of care outlined in national cancer survivorship standards, our findings emphasize the need for system‐wide interventions to address these variations in referrals for care. In response to the identified gaps in our institution, we have implemented several action items to improve both inpatient and outpatient OT/PT referral and utilization with the least amount of resistance (e.g., insurance authorization).

These include: (1) Building a co‐location OT/PT space near the clinic, (2) improving EPIC tabs to highlight who was and was not referred to PT/OT, (3) increasing education for the clinical team, and (4) automate OT/PT referral process across all service lines for all children with cancer upon admission. Future studies should explore how certain service lines have successfully promoted OT/PT referrals and completion of OT/PT sessions, particularly in the outpatient setting. Such research could inform broader adoption of successful strategies and enhance referrals to and utilization of OT/PT services for this population.

## Author Contributions

**Maria C. Swartz:** conceptualization (equal), data curation (equal), formal analysis (equal), funding acquisition (equal), investigation (equal), methodology (equal), project administration (equal), resources (equal), supervision (equal), validation (equal), visualization (equal), writing – original draft (equal), writing – review and editing (equal). **Shiming Zhang:** data curation (equal), formal analysis (equal), investigation (equal), project administration (equal), resources (equal), software (equal), validation (equal), visualization (equal), writing – original draft (equal), writing – review and editing (equal). **Donna Kelly:** conceptualization (equal), data curation (equal), funding acquisition (equal), investigation (equal), methodology (equal), resources (equal), validation (equal), writing – original draft (equal), writing – review and editing (equal). **Clark R. Andersen:** conceptualization (equal), data curation (equal), formal analysis (equal), funding acquisition (equal), investigation (equal), methodology (equal), resources (equal), software (equal), supervision (equal), validation (equal), visualization (equal), writing – original draft (equal), writing – review and editing (equal). **Keri Schadler:** conceptualization (equal), funding acquisition (equal), methodology (equal), resources (equal), validation (equal), writing – original draft (equal), writing – review and editing (equal). **Alakh P. Rajan:** data curation (equal), project administration (equal), visualization (equal), writing – review and editing (equal). **Eduardo Gonzalez Villarreal:** data curation (equal), project administration (equal), writing – original draft (equal), writing – review and editing (equal). **Stephanie J. Wells:** data curation (equal), project administration (equal), resources (equal), writing – review and editing (equal). **Amy Heaton:** project administration (equal), visualization (equal), writing – review and editing (equal). **Karen Moody:** conceptualization (equal), data curation (equal), formal analysis (equal), funding acquisition (equal), investigation (equal), methodology (equal), project administration (equal), resources (equal), supervision (equal), validation (equal), visualization (equal), writing – original draft (equal), writing – review and editing (equal).

## Funding

This work was supported by NIH/NCI (P30CA016672‐454S), University of Texas MD Anderson Cancer Center, UTHealth Houston‐Cancer Prevention Research Institute of Texas Innovation for Cancer Prevention Research (RP210042).

## Ethics Statement

This retrospective study utilized existing electronic medical record data collected for non‐research purposes. The study protocol received ethical approval from The University of Texas MD Anderson Cancer Center IRB (IRB #2021–0820) on October 7, 2021. Given the retrospective nature of the study and the use of electronic medical record data, informed consent from individual patients was waived by the ethics committee in accordance with regulatory guidelines. All patient data were handled confidentially and anonymized to protect privacy and comply with applicable data protection laws and institutional policies.

## Conflicts of Interest

The authors declare no conflicts of interest.

## Supporting information


**Data S1:** Supplementary Information.

## Data Availability

In accordance with institutional policy, raw data will not be made publicly available.
