# Supplementary material for: Barriers to Occupational and Physical Therapy Utilization for Children With Cancer
Source: Cancer Med. 2026 Mar 19;15(3):e71598. doi: 10.1002/cam4.71598 (PMC13093578; doi:10.1002/cam4.71598)
Supplement: Supplementary file 1 — Data S1: Supplementary Information. [file CAM4-15-e71598-s001.docx]

**SUPPLEMENTAL MATERIALS**

**Title:** Barriers to Occupational and Physical Therapy Utilization for Children with Cancer

**Supplement Tables 1-6**

**Supplement Table 1. Variables of interest by level with rationale**

| **Level** | **Variable category** | **Specific variables** | **Rationale** |
| --- | --- | --- | --- |
| Individual | Demographics | Sex, race/ethnicity, language | Individual characteristics that may affect referral (access) and ability to navigate the system for utilization |
| Disease/clinical | Cancer diagnosis | Cancer types | Diagnosis may affect function differently, and may affect referral practice among the clinical team |
| Disease/clinical | Treatment type | Chemotherapy, radiation, hormonal therapy | Treatment intensity may also affect function differently, and may affect referral practice |
| Disease/clinical | Functional indicators | Surgeries (e.g., cancer-related amputation) comorbidities | Direct clinical indicators of rehabilitation needs |
| System/Community | Care setting | Inpatient versus Outpatient | Settings may have different barriers to access and utilization |
| System/Community | Insurance | Type (e.g., private, Medicaid, other) | Insurance authorization may affect access and utilization |
| System/Community | Social Vulnerability Index | Social Vulnerability Index percentile | Neighborhood resources may affect family’s ability to access |

**Supplemental Table 2.** Outpatient and Inpatient OT and PT Referrals and Completion

| Inpatient OT Referral | N | % |  | Inpatient OT Referrals Completed | N | % |
| --- | --- | --- | --- | --- | --- | --- |
| No | 742 | 68.7 |  | No | 67 | 17.8 |
| Yes | 338 | 31.3 |  | Yes | 309 | 82.2 |
|  |  |  |  |  |  |  |
| Inpatient PT Referral | N | % |  | Inpatient PT Referrals Completed | N | % |
| No | 711 | 65.8 |  | No | 42 | 11.2 |
| Yes | 369 | 34.2 |  | Yes | 334 | 88.8 |
|  |  |  |  |  |  |  |
| Inpatient OT or PT Referral | N | % |  | Inpatient OT or PT Referrals Completed | N | % |
| No | 704 | 65.2 |  | No | 24 | 6.4 |
| Yes | 376 | 34.8 |  | Yes | 352 | 93.6 |
|  |  |  |  |  |  |  |
| Outpatient OT Referral | N | % |  | Outpatient OT Referrals Completed | N | % |
| No | 928 | 85.9 |  | No | 168 | 69.1 |
| Yes | 152 | 14.1 |  | Yes | 172 | 30.9 |
|  |  |  |  |  |  |  |
| Outpatient PT Referral | N | % |  | Outpatient PT Referrals Completed | N | % |
| No | 848 | 78.5 |  | No | 101 | 43.5 |
| Yes | 232 | 21.5 |  | Yes | 131 | 56.5 |
|  |  |  |  |  |  |  |
| Outpatient OT or PT Referral |  |  |  | Outpatient OT or PT Referrals Completed | N | % |
| No | 837 | 77.5 |  | No | 95 | 39.1 |
| Yes | 243 | 22.5 |  | Yes | 148 | 60.9 |

**Supplemental Table 3:** Univariate Analysis of Factors Associated with Inpatient Referrals to Occupational or Physical Therapy

| **Covariate** | **Contrast** | **Odds Ratio (95% CI)** | **p*** |
| --- | --- | --- | --- |
| Age, continuous | Unit Change | 0.974 (0.948-1.001) | 0.056 |
| Age, discrete | 10-13 years / 0-9 years | 1.059 (0.677-1.657) | 0.97 |
|  | 14-17 years / 0-9 years | 1.051 (0.734-1.505) | 0.97 |
|  | ≥18 years / 0-9 years | 0.227 (0.125-0.412) | **<0.0001** |
| Sex | Male / Female | 1.200 (0.885-1.627) | 0.24 |
| Language | Spanish / English | 1.901 (1.047-3.450) | 0.066 |
|  | Other / English | 0.842 (0.569-1.246) | 0.59 |
| Race/Ethnicity | Hispanic / Non-Hispanic White | 1.135 (0.783-1.647) | 0.87 |
|  | Non-Hispanic Black / Non-Hispanic White | 1.026 (0.571-1.846) | 1 |
|  | Non-Hispanic Asian / Non-Hispanic White | 0.657 (0.379-1.140) | 0.37 |
|  | (Other/Unknown/Declined) / Non-Hispanic White | 1.075 (0.674-1.715) | 0.98 |
| Primary Insurance Coverage | Private / Medicaid | 0.871 (0.600-1.265) | 0.68 |
|  | (Nonprivate/Other/Government-Issued/Unknown) / Medicaid | 0.879 (0.565-1.368) | 0.78 |
| Chemotherapy Plan | Yes / No | 3.224 (0.826-12.590) | 0.09 |
| Hormonal Therapy Plan | Yes / No | 1.372 (0.715-2.630) | 0.34 |
| Radiation Therapy Plan | Yes / No | 2.247 (1.524-3.314) | **<0.0001** |
| Cancer Type | NNST / (Leukemia/Lymphoma) | 1.119 (0.786-1.592) | 0.84 |
|  | CNS Tumor / (Leukemia/Lymphoma) | 1.129 (0.716-1.780) | 0.89 |
|  | Adult-Type Cancer / (Leukemia/Lymphoma) | 0.345 (0.175-0.682) | **0.006** |
| Procedure | Diagnostic Procedures / None | 1.194 (0.785-1.817) | 0.83 |
|  | (Placement or Removal of Hardware/Medical Devices) / None | 1.660 (1.007-2.736) | 0.18 |
|  | (Other Non–Cancer-Related Surgeries/Procedures) / None | 0.717 (0.438-1.174) | 0.53 |
|  | Amputations and Tumor Resections / None | 1.119 (0.479-2.616) | 0.99 |
|  | Bone Marrow Transplant / None | 1.076 (0.284-4.070) | 1 |
| Procedure – Diagnostic Procedures | Yes / No | 1.158 (0.774-1.732) | 0.48 |
| Procedure – Placement or Removal of Hardware/Medical Devices | Yes / No | 1.665 (1.026-2.701) | **0.039** |
| Procedure – Other Non–Cancer-Related Surgeries/Procedures | Yes / No | 0.645 (0.401-1.039) | 0.07 |
| Procedure – Amputations and Tumor Resections | Yes / No | 1.060 (0.458-2.452) | 0.89 |
| Procedure – Bone Marrow Transplant | Yes / No | 1.018 (0.271-3.823) | 0.98 |
| SVI Overall Percentile | Unit Change | 1.005 (0.999-1.011) | 0.09 |
| Any Comorbidity | Yes / No | 3.039 (1.898-4.865) | **<0.0001** |
| Comorbidities – Conditions Affecting Mobility and Function | Yes / No | 2.255 (1.651-3.080) | **<0.0001** |
| Comorbidities – Acute and Chronic Pain Conditions | Yes / No | 2.871 (2.097-3.931) | **<0.0001** |
| Comorbidities – Cardiopulmonary Diseases | Yes / No | 3.356 (2.428-4.638) | **<0.0001** |
| Comorbidities – Diseases and Conditions Affecting Other Organ Systems | Yes / No | 2.017 (1.454-2.798) | **<0.0001** |

Abbreviations: NNST = non-neural solid tumor, CNS = central nervous system, SVI = Social Vulnerability Index.

*Boldface indicates statistical significance.

**Supplemental Table 4: Univariate Analysis of Factors Associated with Completion of Inpatient Occupational or Physical Therapy Services**

| **Covariate** | **Contrast** | **Odds Ratio (95% CI)** | **p*** |
| --- | --- | --- | --- |
| Age, continuous | Unit Change | 1.014 (0.940-1.094) | 0.71 |
| Age, discrete) | 10-13 years / 0-9 years | 0.658 (0.229-1.890) | 0.75 |
|  | 14-17 years / 0-9 years | 1.012 (0.387-2.641) | 1 |
|  | ≥18 years / 0-9 years | Undefined | 1 |
| Sex | Male / Female | 1.032 (0.446-2.387) | 0.94 |
| Language | Spanish / English | 0.844 (0.237-3.008) | 0.94 |
|  | Other / English | 1.406 (0.401-4.928) | 0.8 |
| Race/Ethnicity | Hispanic / Non-Hispanic White | 1.021 (0.397-2.629) | 1 |
|  | Non-Hispanic Black / Non-Hispanic White | 2.279 (0.283-18.346) | 0.81 |
|  | Non-Hispanic Asian / Non-Hispanic White | Undefined | 1 |
|  | (Other/Unknown/Declined) / Non-Hispanic White | 1.002 (0.305-3.288) | 1 |
| Primary Insurance Coverage | Private / Medicaid | 1.361 (0.510-3.629) | 0.75 |
|  | (Nonprivate/Other/Government-Issued/Unknown) / Medicaid | 1.059 (0.341-3.287) | 0.99 |
| Chemotherapy Plan | Yes / No | Undefined | 0.99 |
| Hormonal Therapy Plan | Yes / No | 8320057.031 (0-∞) | 0.99 |
| Radiation Therapy Plan | Yes / No | 1.508 (0.548-4.148) | 0.43 |
| Cancer Type | NNST / (Leukemia/Lymphoma) | 0.732 (0.267-2.008) | 0.85 |
|  | CNS Tumor / (Leukemia/Lymphoma) | 0.675 (0.198-2.301) | 0.84 |
|  | Adult-Type Cancer / (Leukemia/Lymphoma) | 0.696 (0.078-6.245) | 0.96 |
| Procedure | Diagnostic Procedures / None | 2.661 (0.593-11.950) | 0.56 |
|  | (Placement or Removal of Hardware/Medical Devices) / None | 1.008 (0.320-3.172) | 1 |
|  | (Other Non–Cancer-Related Surgeries/Procedures) / None | 2.742 (0.351-21.449) | 0.76 |
|  | Amputations and Tumor Resections / None | 0.968 (0.118-7.956) | 1 |
|  | Bone Marrow Transplant / None | 0.323 (0.034-3.072) | 0.74 |
| Procedure – Diagnostic Procedures | Yes / No | 2.538 (0.582-11.063) | 0.21 |
| Procedure – Placement or Removal of Hardware/Medical Devices | Yes / No | 0.828 (0.272-2.523) | 0.74 |
| Procedure – Other Non–Cancer-Related Surgeries/Procedures | Yes / No | 2.459 (0.322-18.783) | 0.39 |
| Procedure – Amputations and Tumor Resections | Yes / No | 0.812 (0.101-6.519) | 0.84 |
| Procedure – Bone Marrow Transplant | Yes / No | 0.264 (0.028-2.463) | 0.24 |
| SVI Overall Percentile | Unit Change | 1.007 (0.991-1.024) | 0.37 |
| Any Comorbidity | Yes / No | 5.018 (1.805-13.947) | **0.002** |
| Comorbidities – Conditions Affecting Mobility and Function | Yes / No | 1.114 (0.479-2.593) | 0.8 |
| Comorbidities – Acute and Chronic Pain Conditions | Yes / No | 0.883 (0.353-2.205) | 0.79 |
| Comorbidities – Cardiopulmonary Diseases | Yes / No | 2.589 (1.070-6.267) | **0.035** |
| Comorbidities – Diseases and Conditions Affecting Other Organ Systems | Yes / No | 0.815 (0.294-2.258) | 0.69 |

Abbreviations: NNST = non-neural solid tumor, CNS = central nervous system, SVI = Social Vulnerability Index.

*Boldface indicates statistical significance.

**Supplemental Table 5:** Univariate Analysis of Factors Associated with Outpatient Referrals to Occupational or Physical Therapy

| **Covariate** | **Contrast** | **Odds Ratio (95% CI)** | **p*** |
| --- | --- | --- | --- |
| Age, continuous | Unit Change | 0.986 (0.961-1.011) | 0.26 |
| Age, discrete | 10-13 years / 0-9 years | 1.116 (0.749-1.662) | 0.88 |
|  | 14-17 years / 0-9 years | 0.846 (0.601-1.191) | 0.64 |
|  | 18 years / 0-9 years | 0.877 (0.517-1.486) | 0.9 |
| Sex | Male / Female | 0.981 (0.737-1.308) | 0.9 |
| Language | Spanish / English | 0.757 (0.444-1.292) | 0.49 |
|  | Other / English | 1.723 (1.197-2.481) | **0.007** |
| Race/Ethnicity | Hispanic / Non-Hispanic White | 1.220 (0.856-1.740) | 0.62 |
|  | Non-Hispanic Black / Non-Hispanic White | 1.030 (0.584-1.816) | 1 |
|  | Non-Hispanic Asian / Non-Hispanic White | 1.519 (0.900-2.564) | 0.33 |
|  | (Other/Unknown/Declined) / Non-Hispanic White | 1.879 (1.230-2.872) | **0.013** |
| Primary Insurance Coverage | Private / Medicaid | 1.023 (0.726-1.443) | 0.98 |
|  | (Nonprivate/Other/Government-Issued/Unknown) / Medicaid | 1.509 (1.005-2.265) | 0.09 |
| Chemotherapy Plan | Yes / No | 1.229 (0.392-3.850) | 0.72 |
| Hormonal Therapy Plan | Yes / No | 1.670 (0.916-3.043) | 0.09 |
| Radiation Therapy Plan | Yes / No | 1.288 (0.933-1.779) | 0.12 |
| Cancer Type | NNST / (Leukemia/Lymphoma) | 1.018 (0.714-1.453) | 1 |
|  | CNS Tumor / (Leukemia/Lymphoma) | 0.974 (0.660-1.437) | 0.99 |
|  | Adult-Type Cancer / (Leukemia/Lymphoma) | 0.189 (0.066-0.539) | **0.005** |
| Procedure | Diagnostic Procedures / None | 2.181 (1.452-3.277) | **0.0008** |
|  | (Placement or Removal of Hardware/Medical Devices) / None | 1.508 (0.932-2.442) | 0.32 |
|  | (Other Non–Cancer-Related Surgeries/Procedures) / None | 1.097 (0.709-1.695) | 0.97 |
|  | Amputations and Tumor Resections / None | 1.569 (0.738-3.336) | 0.63 |
|  | Bone Marrow Transplant / None | 0 (0-∞) | 1 |
| Procedure – Diagnostic Procedures | Yes / No | 2.039 (1.379-3.014) | **0.0004** |
| Procedure – Placement or Removal of Hardware/Medical Devices | Yes / No | 1.308 (0.820-2.086) | 0.26 |
| Procedure – Other Non–Cancer-Related Surgeries/Procedures | Yes / No | 0.915 (0.602-1.392) | 0.68 |
| Procedure – Amputations and Tumor Resections | Yes / No | 1.339 (0.636-2.816) | 0.44 |
| Procedure – Bone Marrow Transplant | Yes / No | 0 (0-∞) | 0.97 |
| SVI Overall Percentile | Unit Change | 1.001 (0.995-1.006) | 0.8 |
| Any Comorbidity | Yes / No | 8.785 (5.503-14.026) | **<0.0001** |
| Comorbidities – Conditions Affecting Mobility and Function | Yes / No | 6.409 (4.699-8.742) | **<0.0001** |
| Comorbidities – Acute and Chronic Pain Conditions | Yes / No | 3.870 (2.869-5.220) | **<0.0001** |
| Comorbidities – Cardiopulmonary Diseases | Yes / No | 3.415 (2.534-4.604) | **<0.0001** |
| Comorbidities – Diseases and Conditions Affecting Other Organ Systems | Yes / No | 4.411 (3.198-6.084) | **<0.0001** |

Abbreviations: NNST = non-neural solid tumor, CNS = central nervous system, SVI = Social Vulnerability Index.

*Boldface indicates statistical significance.

**Supplement Table 6: Univariate Analysis of Factors Associated with Completion of Outpatient Occupational or Physical Therapy Services**

| **Covariate** | **Contrast** | **Odds Ratio (95% CI)** | **p*** |
| --- | --- | --- | --- |
| Age, continuous | Unit Change | 1.006 (0.960-1.054) | 0.80 |
| Age, discrete | 10-13 years / 0-9 years | 1.045 (0.515-2.120) | 0.99 |
|  | 14-17 years / 0-9 years | 1.260 (0.672-2.361) | 0.79 |
|  | ≥18 years / 0-9 years | 0.739 (0.289-1.891) | 0.83 |
| Sex | Male / Female | 0.568 (0.335-0.962) | **0.035** |
| Language | Spanish / English | 0.503 (0.189-1.339) | 0.29 |
|  | Other / English | 1.158 (0.612-2.191) | 0.85 |
| Race/Ethnicity | Hispanic / Non-Hispanic White | 0.567 (0.295-1.091) | 0.27 |
|  | Non-Hispanic Black / Non-Hispanic White | 0.931 (0.316-2.745) | 1 |
|  | Non-Hispanic Asian / Non-Hispanic White | 0.776 (0.302-1.995) | 0.92 |
|  | (Other/Unknown/Declined) / Non-Hispanic White | 0.532 (0.253-1.118) | 0.28 |
| Primary Insurance Coverage | Private / Medicaid | 1.795 (0.962-3.350) | 0.12 |
|  | (Nonprivate/Other/Government-Issued/Unknown) / Medicaid | 1.309 (0.643-2.664) | 0.67 |
| Chemotherapy Plan | Yes / No | 0.506 (0.052-4.938) | 0.56 |
| Hormonal Therapy Plan | Yes / No | 4.070 (1.159-14.284) | **0.028** |
| Radiation Therapy Plan | Yes / No | 0.965 (0.544-1.712) | 0.9 |
| Cancer Type | NNST / (Leukemia/Lymphoma) | 3.111 (1.628-5.943) | **0.002** |
|  | CNS Tumor / (Leukemia/Lymphoma) | 2.126 (1.063-4.254) | 0.09 |
|  | Adult-Type Cancer / (Leukemia/Lymphoma) | 1.286 (0.170-9.704) | 0.98 |
| Procedure | Diagnostic Procedures / None | 0.746 (0.379-1.470) | 0.78 |
|  | Placement or Removal of Hardware/Medical Devices / None | 0.995 (0.428-2.316) | 1 |
|  | (Other Non–Cancer-Related Surgeries/Procedures) / None | 2.053 (0.856-4.921) | 0.31 |
|  | Amputations and Tumor Resections / None | 2.737 (0.559-13.409) | 0.53 |
| Procedure – Diagnostic Procedures | Yes / No | 0.642 (0.336-1.226) | 0.18 |
| Procedure – Placement or Removal of Hardware/Medical Devices | Yes / No | 0.926 (0.410-2.091) | 0.85 |
| Procedure – Other Non–Cancer-Related Surgeries/Procedures | Yes / No | 2.105 (0.903-4.904) | 0.08 |
| Procedure – Amputations and Tumor Resections | Yes / No | 2.657 (0.552-12.792) | 0.22 |
| SVI Overall Percentile | Unit Change | 0.992 (0.981-1.002) | 0.10 |
| Any Comorbidity | Yes / No | 0.756 (0.293-1.947) | 0.56 |
| Comorbidities – Conditions Affecting Mobility and Function | Yes / No | 2.308 (1.341-3.971) | **0.003** |
| Comorbidities – Acute and Chronic Pain Conditions | Yes / No | 1.473 (0.867-2.503) | 0.15 |
| Comorbidities – Cardiopulmonary Diseases | Yes / No | 0.912 (0.544-1.529) | 0.73 |
| Comorbidities – Diseases and Conditions Affecting Other Organ Systems | Yes / No | 1.101 (0.610-1.990) | 0.75 |

Abbreviations: NNST = non-neural solid tumor, CNS = central nervous system, SVI = Social Vulnerability Index.

*Boldface indicates statistical significance.
